# Proof of Concept: Matrix metalloproteinase inhibitor decreases inflammation and improves muscle insulin sensitivity in people with type 2 diabetes

**DOI:** 10.1186/1476-9255-9-35

**Published:** 2012-10-01

**Authors:** Karen Frankwich, Courtney Tibble, Moises Torres-Gonzalez, Mariah Bonner, Roy Lefkowitz, Matt Tyndall, Geert W Schmid-Schönbein, Francisco Villarreal, Mike Heller, Karen Herbst

**Affiliations:** 1 Veteran’s Affairs, San Diego Healthcare System, 3350 La Jolla Village Drive (111 G), San Diego, CA, 92161, USA; 2 Department of Medicine, Division of Endocrinology & Metabolism, University of California, 9500 Gilman Drive, #0673 La Jolla, San Diego, CA, 92093, USA; 3 Department of Bioengineering, University of California, 9500 Gilman Drive, #0412 La Jolla, San Diego, CA, 92093, USA; 4 Department of Pharmacology, University of California, 9500 Gilman Drive, #0636 La Jolla, San Diego, CA, 92093, USA; 5 Veteran’s Medical Research Foundation, 3350 La Jolla Village Drive (111 G), San Diego, CA, 92161, USA

**Keywords:** Diabetes, Doxycycline, Insulin sensitivity, Matrix metalloproteinases, Myeloperoxidase

## Abstract

**Background:**

Obesity is a state of subclinical inflammation resulting in loss of function of insulin receptors and decreased insulin sensitivity. Inhibition of the inflammatory enzymes, matrix metalloproteinases (MMPs), for 6 months in rodent models restores insulin receptor function and insulin sensitivity.

**Methods:**

This 12-week double-blind, randomized, placebo (PL)-controlled proof-of-concept study was performed to determine if the MMP inhibitor (MMPI), doxycycline, decreased global markers of inflammation and enhanced muscle insulin sensitivity in obese people with type 2 diabetes (DM2). The study included non-DM2 controls (n = 15), and DM2 subjects randomized to PL (n = 13) or doxycycline 100 mg twice daily (MMPI; n = 11). All participants were evaluated on Day 1; MMPI and PL groups were also evaluated after 84 days of treatment.

**Results:**

There was a significant decrease in inflammatory markers C-reactive protein (P < 0.05) and myeloperoxidase (P = 0.01) in the MMPI but not PL group. The MMPI also significantly increased skeletal muscle activated/total insulin signaling mediators: 3’phosphoinositide kinase-1 (PDK1) (p < 0.03), protein kinase B (PKB/Akt) (p < 0.004), and glycogen synthase kinase 3ß (GSK3ß) (p < 0.03).

**Conclusions:**

This study demonstrated short term treatment of people with diabetes with an MMPI resulted in decreased inflammation and improved insulin sensitivity. Larger, longer studies are warranted to determine if doxycycline can improve glucose control in people with diabetes.

**Trial Registration:**

Clinicaltrials.gov NCT01375491

## Background

As early as 1993, obesity was recognized as a heightened state of inflammation with inflammatory cells increasing within enlarging adipose tissue depots [1]. As part of inflammation, expression levels and activity of matrix metalloproteinases (MMPs), a family of extracellular endopeptidases, are significantly elevated in rodent models of obesity [2] and obese humans. [3-5] MMPs, ubiquitously found in tissue and secreted by immune cells, cleave extra-cellular matrix components of body tissues and other proteins during wound repair, tissue remodeling, cancer growth and metastases, and inflammation. MMPs reported to be elevated in obesity compared to healthy control populations include MMP-2, -3,-8 and −9, with less elevation of MMP-7 [3][6], the latter thought to be a marker of adipocyte maturity [7].

In animal models of diabetes and hypertension, excessive proteolytic MMP activity cleaves the extracellular domain of the insulin receptor, as well as other receptors, resulting in loss of function, including a reduction in insulin sensitivity. [8,9] Blockade of proteases by an MMP inhibitor (MMPI) therefore improved not only MMP levels and activity but blood glucose levels by 33% in mice. [9] Regulation of MMPs is, however, complex, where transcriptional regulators and tissue inhibitors of MMP (TIMPs) modulate MMP levels and activity, respectively. Only examining MMP levels may therefore, not provide complete information. For example, MMP-2 and MMP-9 decreased after exercise-induced weight loss [10,11] but in a separate study, MMP-2 and MMP-3 levels did not decline after bariatric surgery. [7] Clinical outcome studies are therefore warranted to determine whether MMP inhibition reduces inflammation and improves insulin sensitivity in obese diabetic patients.

Doxycycline, a synthetic derivative of tetracycline, is a broad spectrum inhibitor of MMP-1, -2, -3, -8, -9, -10, and −13 [9,12-15]. Doxycycline has been used successfully as an MMPI in a number of pathological states in which MMPs are known to be elevated. For example, doxycycline significantly reduced progressive aortic degeneration after aneurysm repair along with MMP-9 levels in human subjects after 6 months of treatment [16]. Doxycycline was also more effective than a beta-blocker at preventing thoracic aortic aneurysm development in Marfan syndrome through inhibition of MMP-2 and −9 [15]. Golub et al. also demonstrated inhibition of MMP activity in rodent models of DM2 by MMPIs of which doxycycline was the most potent. [14,17]

A means to determine if MMPI translates into improved insulin sensitivity in diabetic patients is to assess the activity of molecules in the insulin signaling pathway in skeletal muscle. Insulin binding to its receptor activates phosphatidyl inositol-3-kinase (PI3K) resulting in recruitment and phosphorylation of PDK1 (pPDK1), which in turn phosphorylates and activates Akt (pAkt). [18] pAkt triggers an increase in insulin responsive glucose transporter (GLUT4) translocation and recruitment to the cell membrane, increasing glucose uptake by the cell. pAkt also phosphorylates and thus *in*activates GSK3β (pGSK3β). This prevents GSK3β from phosphorylating (and thus *in*activating) glycogen synthase – which leaves the glycogen synthase active to continue glycogen production. Enhancement of insulin sensitivity in muscle, therefore, should increase levels of pPDK1, pAkt and pGSK3β.

In a proof of concept clinical trial we determined whether doxycycline (MMPI) can reduce inflammation and improve insulin sensitivity. For this purpose, we enrolled DM2 patients into a double-blind randomized, short-term, clinical trial to receive 84 days of either MMPI or PL. Markers of inflammation and muscle insulin signaling mediators were measured before and after treatment and compared to placebo controls.

## Methods

### Eligibility and exclusion criteria

Participants were eligible if they had DM2 <10 years with A1c 7.5-10%, no or stable retinopathy, and normal creatinine. Because this was a proof of concept study, participants could use any hypoglycemic medication including insulin but not thiazolidinediones, which are known to inhibit MMPs [19]. Using published data on relative MMP-9 levels [20], we estimated a power greater than 99% at a two-sided alpha of 5% to see a difference in mean C-reactive protein (CRP) and MMP levels between DM groups with 15 subjects per group.

### Study design

This study was approved by the University of California, San Diego (UCSD) Human Research Protection Program and the Research and Development Committee at the Veterans Affairs San Diego Healthcare System which support research that is in compliance with the Helsinki Declaration as part of the United States’ Office for Human Research Protections. All participants completed an informed consent process prior to enrollment. The study was a double-blind, randomized, PL-controlled trial of the oral antibiotic, doxycycline, compared to PL in obese patients with DM2. The participants with DM2 were randomly assigned to either 100 mg doxycycline (MMPI) twice daily or PL until day 84 (D84). The main inflammatory outcome measures, CRP and myeloperoxidase (MPO) levels, were measured at baseline (day 1; D1) and D84. Muscle biopsies for total and phosphorylated PDK1, Akt, and GSK3β were obtained for 7 consecutive participants in the PL and MMPI groups on D1 and D84, directly after a 180-minute oral glucose tolerance test (OGTT; a relative insulin-stimulated state). We did not expect body composition changes in this short study but completed dual-energy x-ray absorptiometry (DXA) scans on D1 and D84 to confirm that any changes in muscle insulin sensitivity were not due to changes in body composition. While we did not expect change in global measures of glucose homeostasis in this short proof of concept study, based on the much longer time needed to see changes in these parameters in rodent studies, [9] we did assess hemoglobin A1c (A1c), calculated indices from the OGTT and used calculations from the homeostasis model assessment (HOMA) on D1 and D84 as comparators for future studies. Additional inflammatory markers measured on D1 and D84 included a cytokine array. Creatinine levels, the liver enzyme, AST, and a lipid panel as safety labs, and vital signs were measured on D1 and D84. Healthy control participants (controls), enrolled to provide comparison data for people without DM2, underwent screening and D1 visits only.

### Muscle biopsies

The skin was sterilely prepped and draped over the vastus lateralis 7 cm caudal to the medial patellar line. Lidocaine (10 cc) was infused superficially then deep and the skin incised 5 mm with a scalpel. A trochanter was used to obtain 400 mg of muscle that was immediately flash frozen in liquid nitrogen. Cyanoacrylates were used to close the wound followed by firm pressure and a compression wrap.

### MMP level and activity

Plasma samples from the OGTT time zero were mixed for 30 min with 0.5 mg/mL of a charge-changing peptide substrate for MMP-2 and MMP-9 (Ac-NGDPVGLTAGAGK-NH2 [synthesized by Aapptec (Louisville, KY) and labeled with Bodipy FL-SE (Invitrogen)]) then loaded into 20% polyacrylamide gels and electrophoresed for 10 minutes prior to imaging as previously described. [21] A global assay for MMP activity levels was also performed on plasma samples using the SensoLyte®520 Generic MMP assay kit according to manufacturer’s instructions (#71158; AnaSpec, San Jose, CA.).

### Western immunoblotting

Total proteins were extracted from muscle biopsies of 7 consecutive PL and 7 MMPI participants, in the presence of a mix of protease and phosphatase inhibitors (Thermo Scientific, Waltham, MA), which were separated by SDS-polyacrylamide gel electrophoresis (NuPAGE 4–12% polyacrylamide Bis-Tris, Invitrogen) and transferred to PVDF membranes. Primary antibodies used for immunoblotting included total Akt and phospho Akt(Thr308); total PDK1 and phospho PDK1; total GSK3β and phospho GSK3β; and GAPDH (Cell Signaling Technology, Danvers, MA.). Incubation of the immunoblots with horseradish peroxidase-conjugated secondary antibodies was carried out after membranes were incubated with their corresponding primary antibodies. The immunoblots were detected with Western lightning enhanced chemiluminescence detection reagent (PerkinElmer LAS, Inc., Norton, OH). Band intensity was quantified with Scion ImageJ Software for personal computers (Scion, Frederick, MD) and expressed in arbitrary units. Day 84 values were normalized relative to baseline values equal to 1. GAPDH was used as an internal standard.

### Oral glucose tolerance test (OGTT)

The OGTT was performed once in all participants on D1 and on D84 in subjects with DM2 according to published guidelines. [22] Following an overnight fast, 75 grams of liquid carbohydrate was administered over a span of 5 minutes or less, with blood samples obtained via heparin lock at −20, -10, 0, 15, 30, 60, 90, 120, and 180 min to determine glucose concentrations using a glucose analyzer (YSI Life Sciences, Yellow Spring, OH) and insulin levels. Plasma insulin levels were measured on stored frozen (−80°C) samples by double-antibody sandwich electro chemiluminescence immunoassay (ECLIA; cat#L45CA-1; Meso Scale Diagnostics [MSD], Gaithersburg, MD, USA) on Sector Imager 2400 (SI2400; MSD, Gaithersburg, MD, USA) by the UCSD Core laboratory. Data was analyzed for measures of insulin sensitivity and glucose utilization using published methods. [23]

### Glucose homeostasis

Homeostasis Model Assessment of insulin secretion (HOMA-IS) was calculated as $\left[20\times \left(\text{fasting insulin}/7.175\right)/\left(\text{fasting glucose}-3.5\right)\right]$[24,25] and HOMA-IR as $\left[\text{fasting insulin}\;\left(\text{pmol}/\text{l}\right)\times \text{fasting glucose}\left(\text{mmol}/\text{l}\right)\right]/135.$ The early secretory response of insulin to an oral glucose load (Δinsulin30–0/Δglucose30–0) was calculated as [Δ insulin (30 min − 0 min)]/[Δ glucose (30 min − 0 min)] [26]. The incremental (above the baseline value) area under the curve (AUC_i_) for glucose and insulin was calculated using GRAPHPAD 5.0.

### Dual X-ray absorptiometry (DXA)

Fat and fat-free mass were measured by DXA scan for whole-body composition on a Hologic Discovery W (Boston, MA) in the UCSD Clinical Trials and Research Institute (CTRI) and analyzed using software QDR DICOM for Windows XP.

### Other laboratory methods

Serum AST, creatinine, total cholesterol, triglycerides, HDL, LDL, and A1c were measured at UCSD laboratories using established procedures.

### Biomarkers

Assays were performed on plasma samples stored in −80 degree refrigerators according to manufacturer’s kit instructions. MPO ELISA assay (#30-6613A; ALPCO, Salem, NH), serum cytokines (Millipore, Billerica, MA), CRP ELISA (#DCRP00; R&D Systems, Inc., Minneapolis, MN).

### Statistical methods

Randomization to PL or MMPI was in blocks of two using Research Randomizer. [27] Data are presented as average ± SEM. Paired t-tests for pre- and post-treatment values, t-tests between the PL and MMPI groups, and AUC for insulin and glucose values obtained from the OGTT were performed using commercial software (GraphPad Prism version 5.00, GraphPad Software, San Diego California USA, http://www.graphpad.com). For MMP 2/9 activity analyses, a Wilcoxon signed rank test and sign tests were used for paired comparisons. For 3 group comparisons (control vs. PL D1 vs. MMPI D1 or control vs. PL D84 vs. MMPI D84), a Kruskal-Wallis test was used, followed by post-hoc Mann–Whitney-Wilcoxon tests with a Bonferroni correction (SPSS). Post-hoc analyses were performed in order to determine which pair(s) of groups within that set explained the statistical significance. A p-value of .05 was used as the criterion for declaring statistical significance, with the exception that post-hoc tests used a p-value of 0.02.

## Results

### Study participants

A total of 39 participants enrolled in this study including 15 controls without DM2 whose data served as comparison values for D1 data obtained from participants with DM2. Twenty-four controls were consented, three did not qualify and six failed to show for D1 procedures. Thirty-three participants with DM2 were enrolled, three did not qualify, three were unable to start the study due to medical problems and three failed to show for D1 procedures. Thirteen participants with DM2 were randomized to the PL group and eleven were randomized to the MMPI group. One subject in the PL group dropped out due to a skin yeast infection and one participant dropped from the MMPI group because of sun sensitivity. A second subject developed joint aches, night sweats, nausea and dizziness after MMPI treatment which resolved after stopping the drug. Two patients on MMPI developed nausea but completed the study.

There were no significant differences between baseline characteristics for the DM2 participants (Table 1). Statistically significant differences between the control and DM2 groups were found for weight, BMI, fasting glucose and insulin, and A1c (Table 1).

**Table 1:** Baseline characteristics of study participants (Mean ± SEM)

**Characteristics**	**Control**	**Placebo**	**Doxycycline**
Sample size	15	13	11
Age (years)	48.7 ± 3.1	54.5 ± 1.7	55.3 ± 1.9
Females (%)	5 (0.33)	2 (0.15)	2 (0.18)
Weight (kg)	79.1 ± 3.6	121.6 ± 6.4**	116.8 ± 9.7**
Total Lean Mass (kg)	52.5 ± 17.5	75.5 ± 15.3**	66.7 ± 13.8*
Total Fat Mass (kg)	20.1 ± 5.8	40.7 ± 10.3**	37.8 ± 13.3
BMI (kg/m^2^)	26.8 ± 0.86	39.7 ± 1.4**	37.9 ± 2.6**
Fasting Glucose (mg/dL)	90.8 ± 7.4	178.2 ± 43.8**	149.4 ± 53.4**
Insulin (uIU/mL)	4.0 ± 1.9	41.0 ± 35.1**	39.4 ± 43.2*
A1c (%)	5.4 ± 0.06	8.5 ± 0.4**	8.2 ± 0.5**
**Race or ethnic group no. (%)**			
Non-Hispanic white	13 (87)	13 (100)	9 (82)
Black	1 (7)	0 (0)	1 (9)
Hispanic	1 (7)	0 (0)	1 (9)

*P < 0.05 versus control , **P < 0.001 versus control.

### Markers of inflammation

**Figure 1:**
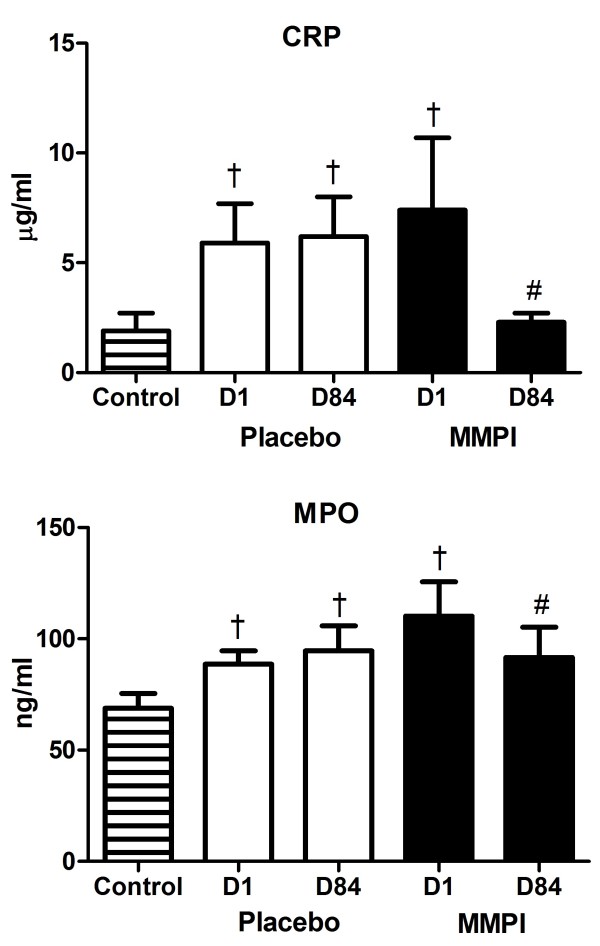
**MPO and CRP levels for the Control, PL, and MMPI groups.** MPO and CRP levels for the Control group at D1, and the PL and MMPI groups at D1 and D84. † P < 0.05 versus Control group. # < 0.05 versus D1 of the MMPI group.

Baseline levels of CRP were higher in the PL and MMPI groups (P < 0.05; Table 2) compared to controls. There was no significant change in CRP in the PL group (P = 0.8) but a significant decrease in CRP in the MMPI group from D1 to D84 (P < 0.05; Table 2; Figure 1). Baseline levels of MPO were significantly higher in the PL and MMPI groups on D1 and in the PL group on D84 compared to controls (Figure 1). MPO in the PL group did not change significantly between D1 and D84 (P = 0.4). MPO significantly decreased in the MMPI group from D1 to D84 (P = 0.001; Table 2; Figure 1). There was no significant change from D1 to D84 for cytokines in the PL or MMPI groups except for a significant decrease in IL-10 in the PL group (P < 0.04; Table 2).

**Table 2:** Outcome measures on D1 and D84 (Mean ± SEM)

		**Placebo Group (n = 11)**		**MMPI Group (n = 9)**	
**Measures**	**Control**	**D1**	**D84**	**D1**	**D84**
CRP	1.9 ± 0.8	5.9 ± 1.8*	6.2 ± 1.8*	7.4 ± 3.3*	2.3 ± 0.4†
MPO	68.8 ± 6.6	88.6 ± 6.0*	94.5 ± 11.3*	110.2 ± 15.3*	91.6 ± 13.6†
SBP (mmHg)	115.7 ± 19.6	127.7 ± 5.7	133.4 ± 4.8	136.2 ± 6.5	137.5 ± 5.8
DBP (mmHg)	72.0 ± 10.5	77.0 ± 10.1	77.8 ± 11	77.8 ± 8.0	77.4 ± 11.2
TC (mg/dL)	177.5 ± 28.0	178.8 ± 13	152.2 ± 6	177.9 ± 14	165.1 ± 10
HDL (mg/dL)	54.0 ± 15.2	40.5 ± 3.2**	36.58 ± 3.0**	38.44 ± 2.6**	38.44 ± 3.2**
LDL (mg/dL)	106.7 ± 22.2	99.6 ± 12	82 ± 7	86 ± 9	86.4 ± 8.8
TG (mg/dL)	84.4 ± 39.0	209.1 ± 30**	191.5 ± 36**	235.9 ± 48**	188.6 ± 37**
AST (IU/L)	22.8 ± 7.7	30.8 ± 4.1	33.0 ± 6.1	24.4 ± 2.9	20.1 ± 1.4
Creatinine (mg/dL)	0.9 ± 0.2	0.83 ± 0.05	0.83 ± 0.07	0.91 ± 0.05	0.92 ± 0.06
Fasting glucose (mmol/L)	5.1 ± 1.2	9.4 ± 0.5**	8.8 ± 0.6**	8.0 ± 0.7**	8.0 ± 1.0**
A1C (%)	5.4 ± 0.2	8.6 ± 0.4	8.0 ± 0.3	8.3 ± 0.6	7.8 ± 0.4
AUC_i_	0.25 ± 0.07	0.57 ± 0.16	0.57 ± 0.13*	0.89 ± 0.31*	0.71 ± 0.29
HOMA-IS	53.4 ± 7.6	135.3 ± 32.7	164 ± 46.8	244.9 ± 98.5	433.1 ± 272.1
HOMA-IR	1.06 ± 0.1	21 ± 5.1	26 ± 9.8	14.3 ± 4.7	23.0 ± 11.1
ΔI30–0/ΔG30–0	98 ± 17.5	37.3 ± 51.1	54.3 ± 30	73.7 ± 35.9	16.6 ± 20.1†
Global MMP (uM 5-FAM/h-ug protein)	47.2 ± 3.2	43.8 ± 4	56.6 ± 10.1	56.8 ± 13.3	59.7 ± 4.3
IL-1β (pg/ml)	0.15 ± 0.06	0.14 ± 0.05	0.13 ± 0.04	0.27 ± 0.15	0.46 ± 0.22
IL-2 (pg/ml)	1.8 ± 0.6	1.1 ± 0.4	0.9 ± 0.3	0.7 ± 0.3	1.1 ± 0.4
IL-4 (pg/ml)	10.0 ± 2.5	7.3 ± 2.1	9.9 ± 4.5	9.8 ± 4.2	7.4 ± 2.3
IL-6 (pg/ml)	6.9 ± 1.5	5.3 ± 1.9	8.4 ± 3.5	10.9 ± 4.3	12.9 ± 5.4
IL-8 (pg/ml)	2.4 ± 0.4	2.8 ± 0.5	3.2 ± 0.5	4.8 ± 0.9	5.8 ± 1.0
IL-10 (pg/ml)	16.6 ± 3.8	15.1 ± 3.5	11.3 ± 3.0†	16.4 ± 5.2	16.8 ± 3.8
TNF-α (pg/ml)	4.7 ± 0.6	4.4 ± 0.4	4.4 ± 0.7	6.6 ± 1.3	5.8 ± 1.1

*P < 0.05 versus control, **P < 0.01 versus control, †P < 0.05 vs. D1.

### Insulin signaling

**Figure 2:**
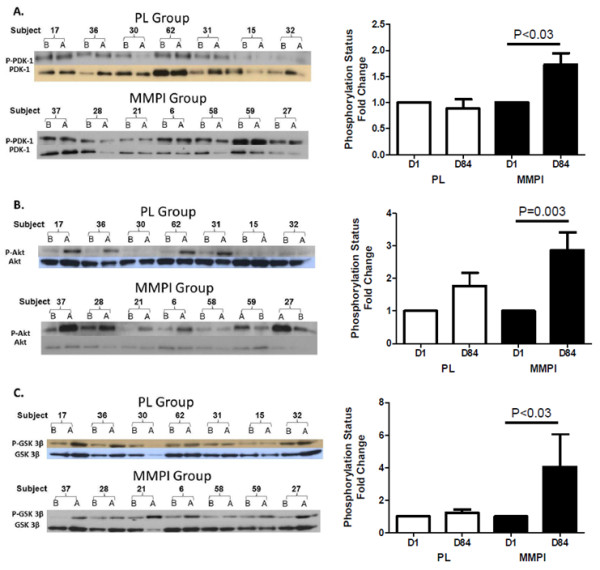
**Activation of muscle insulin signaling mediators in PL and MMPI groups.** MMPI but not PL improves insulin signaling in muscle. Left side panels, **A**, **B** and **C** are digital representations of western immunoblots of key mediators in the insulin signaling pathway (PDK1, Akt, GSK3β. Amounts of phosphorylated and total PDK-1 (**A**), Akt (**B**) and GSK3-β (**C**) in MMPI and PL participants were analyzed at D1 (labeled B on blots) and D84 (labeled A on blots). Specimens for this analysis were obtained from muscle biopsies of seven consecutive age-matched PL and MMPI subjects. In the right side panel, the data is presented as fold change from D1 to D84 in the percentage of phosphorylated (activated) to total molecules in the PL and MMPI groups.

There was no difference between the participants in the PL (n = 7) or MMPI group (n = 7) for age (54.0 ± 7.1y; 56. ±5.3y), weight (120.8 ± 22.7 kg; 113.7 ± 35.2 kg), BMI (40.0 ± 6.2 kg/m^2^; 35.7 ± 7 kg/m^2^) or A1c (9.0 ± 1.5%; 7.7 ± 1.7%) for which muscle was assessed for activation of insulin signaling mediators. In the PL group, there were no statistically significant differences in the phosphorylation status of PDK1, Akt, or GSK3β from D1 to D84 (Figure 2). In contrast, in the MMPI group there was a statistically significant increase in the phosphorylation status of PDK1, Akt, and GSK3β on D84 compared to D1 (Figure 2).

### Vital signs and lipids

There was no significant change in systolic or diastolic blood pressure from D1 to D84 in the PL or MMPI group (Table 2). There was no significant change in any parameter of the lipid profile for the MMPI group (Table 2). In the PL group, total cholesterol decreased significantly (P = 0.015) as did HDL (P = 0.009) from D1 to D84. There was no significant change in triglycerides (P = 0.3) or LDL (P = 0.08) in the PL group from D1 to D84.

### AST and creatinine

There was no significant change in creatinine level in the PL (P = 1.0) or MMPI group (P = 0.7; Table 2) or AST level in the PL (P = 0.6) or MMPI group (P = 0.1; Table 2) from D1 to D84.

### Body composition

There was no significant change in weight in the PL (121.6 ± 6.4 vs. 122.6 ± 6.4 kg) or MMPI group (125.4 ± 12.2 vs. 126.7 ± 12.7 kg; P = 0.2 for both), lean mass in the PL (75.5 ± 15.3 vs. 74.5 ± 14.1 kg) or MMPI group (66.7 ± 13.8 vs. 65.9 ± 14.2 kg; P = 0.8 for both) or fat mass in the PL (40.7 ± 10.3 vs. 41.5 ± 10.1) or MMPI group (37.8 ± 13.3 vs. 39.8 ± 15.1 kg; P = 0.3 for both) between D1 and D84.

### Glucose homeostasis

The fasting glucose level was significantly lower in the control group compared to the MMPI and PL groups on D1 and D84 (Table 2). There was no significant change in A1c between D1 and D84 for the PL or MMPI group (Table 2). The mean percent change in A1c values between the MMPI and PL groups was not significantly different (data on file; P = 0.6). The AUC_i_ did not change from D1 to D84 for the PL or MMPI groups (Table 2). The AUC_i_ for the PL group was not significantly different compared to the control group on D1 but became significantly higher at D84 (Table 2). The AUC_i_ for the MMPI group was significantly higher than the control group at D1 but became non-significantly different at D84 (Table 2). There was no change in the ΔI30–0/ΔG30–0 in the PL group from D1 to D84. The ΔI30–0/ΔG30–0 significantly decreased in the MMPI group from D1 to D84 (Table 2). There was no significant difference for HOMA-IS for the PL group at D1 compared to D84 (P = 0.4; Table 2). The HOMA-IS trended higher for the MMPI group on D84 compared to D1 (P = 0.05; Table 2). There was no significant change in the HOMA-IR for the PL (P = 0.4) or MMPI groups (P = 0.07) from D1 to D84 (Table 2).

### MMP level and activity

There was no difference in global MMP level between the control group and the PL or MMPI groups on D1 or D84, and no change in the global MMP level in the PL or MMPI groups from D1 to D84 (Table 2).

**Figure 3:**
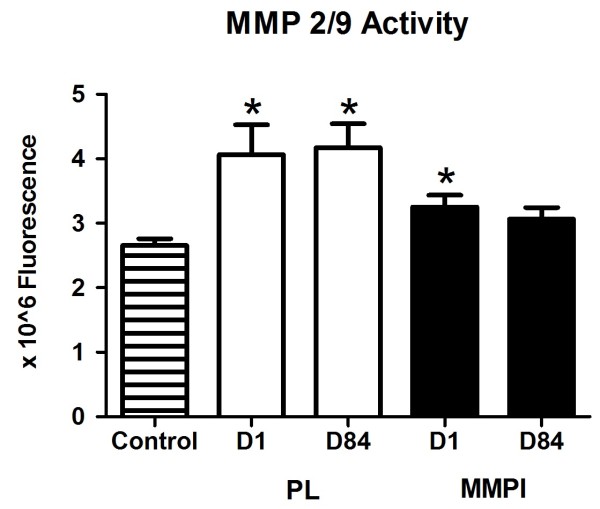
**MMP 2/9 activity in the Control, PL, and MMPI groups.** MMP 2/9 activity in the Control group (D1) and PL and MMPI groups (D1 and D84). Activity was measured after mixing plasma with a charge-changing peptide substrate for MMP-2 and MMP-9 followed by electrophoresis (see Methods). *P = 0.001 versus the control group.

MMP 2/9 activity was significantly lower in the control group compared to the PL and MMPI groups on D1, and PL group on D84 (Figure 3). There was no difference in MMP 2/9 activity between the PL and MMPI groups on D1 or D84 (Figure 3).

## Discussion

This proof of concept study confirmed that in only 12 weeks, the MMPI, doxycycline, improved global measures of inflammation and muscle insulin sensitivity in people with DM2.

Global markers of inflammation, CRP and MPO levels in plasma, were significantly higher in obese participants with diabetes before treatment compared to controls, supporting the pro-inflammatory state expected in this population. CRP is a global measure of inflammation and oxidative stress even in apparently “healthy” populations [28] and MPO is a lysosomal enzyme in neutrophils that has been associated with coronary plaque instability [29] and therefore increased cardiovascular risk. [30] As expected, MPO levels have been shown to be higher in people with diabetes compared to controls. [31] MMPI treatment resulted in a significant decrease in both CRP and MPO by the end of the study whereas PL had no effect, supporting the anti-inflammatory properties of doxycycline. While medications such as HMG coA reductase inhibitors (“statins”) or aspirin are known to affect inflammatory markers (i.e. CRP, MPO), participants did not change medications during the study and so drug effects for our study participants should have been stable. Other than a significant decrease in the anti-inflammatory cytokine IL-10 in the PL group, there were no significant changes in cytokine levels in the PL or MMPI group. Changes in expression of cytokines after MMPI may be better measured in tissues rather than blood.

To test for an effect of MMPI on insulin sensitivity, three different kinases in the insulin signaling cascade in muscle (PDK1, Akt, GSK3β) were examined in muscle biopsies obtained directly after an OGTT (a relative insulin-stimulated state). In the MMPI treated group, there was a significant increase in the phosphorylation status of all of three kinases, indicating an improvement in the insulin sensitivity of the muscle, with no change in the PL group. Data are needed to link the global decrease in inflammation with improved insulin sensitivity in muscle.

Golub et al. first demonstrated inhibition of MMPs by tetracyclines in people with diabetes. [14,17,32] The decision to use doxycycline in this study was based on these data, its broad spectrum MMPI [9,12,13], and its successful MMPI in a number of pathological states [16] including an animal model of diabetes. [9] The literature provides additional evidence that doxycycline can inhibit the activity of other classes of proteases such as plasmin (a serine protease) [33], and act as an oxygen radical scavenger. [34] Doxycycline is also known to be relatively well tolerated with minor side-effects. This study resulted in one case of sun sensitivity with doxycycline use, two cases of nausea and one patient developed serum sickness. Serum sickness like syndrome is known to occur with the use of the tetracyclines, minocycline [35] and doxycycline. [36]

Dosing schemes of MMPI vary widely from 40 mg daily [37] and upwards, including the usual adult antibiotic dose of 100 mg twice daily used in this proof of concept study, a dose reported to reduce CRP levels during inflammation associated with infection [38]. That doxycycline improved insulin sensitivity in muscle but not global glucose homeostasis in people with DM2 suggests the need for larger and longer clinical trials, as well as a dose finding study as lower amounts of doxycycline (40 mg daily) have been shown to significantly reduce CRP in people with cardiovascular disease over 6 months [39].

The proposed mechanism by which doxycycline improves insulin sensitivity, is by inhibition of inflammatory MMPs that cleave the extracellular insulin receptor domain, thereby inactivating the receptor. [9] Doxycycline and other MMPI have been shown to inhibit cleavage of other receptors including vascular endothelial growth factor receptor 2 (VEGFR-2). [40] We did not find a difference in global MMP levels between control participants and those with DM2, and no change in global MMP levels after MMPI; this may be due to the relatively low sensitivity of this technique. Using a new and more sensitive technology to measure MMP 2/9 activity, [21] we found higher MMP 2/9 activity levels in all obese study participants with diabetes compared to the control group prior to treatment, in agreement with others. [2,19,41] This difference is likely due to the higher sensitivity of the electrophoretic zymographic technique and the fact that it does not require separation of blood cells from plasma. [21] However, we found no significant effect of doxycycline on blood levels of MMP 2/9 activity at study completion using this new technique. We hypothesize that although we found improvements in insulin sensitivity, the time of the study was not long enough to see a reduction in these MMP levels in people with severe insulin resistance, and that gene expression of MMPs and TIMP levels need to be measured along with MMPs. During our study, it was reported that blood MMP 2/9 activity levels do not change in participants with DM2 during an OGTT. [42] It appears that MMP blood activity, at present, could be more useful in screening for whether a patient should take MMPI (after observing elevated MMP activity), but isn’t as sensitive for monitoring the effect of treatment after a glucose load. It may require a stronger stimulus than glucose alone, such as a mixed meal, to induce measureable changes in MMP levels in the blood.

There are many factors that can affect MMP activity in blood, in addition to DM2. The development of more specific protease substrates that better differentiate the activity from individual types of MMPs and other proteases would be useful in studies such as these. From animal and *in vitro* studies, it may be that the best way to measure the effect of MMPI on MMP activity is not through blood samples but in tissues such as adipose tissue, where MMP activity is increased in obesity and tissue inhibitors of MMPs (TIMPs) are decreased. [2] Studies are ongoing to test whether MMPI can affect MMP and TIMP levels, and insulin receptor integrity in humans.

Commonly measured clinical parameters of DM control were evaluated in this study including weight, A1c, triglycerides, and blood pressure which did not change in either the MMPI or PL groups. The early secretory response of insulin to an oral glucose load, however, was significantly lower after MMPI treatment as was the secretion of insulin measured by the HOMA-IS, and the AUC_i_ was improving in the MMPI group but not the PL group. While there was a significant increase in activation of molecules in the insulin signaling pathways during this period, the time it takes to see a clinically significant effect on overall glucose control is likely longer. Improvements in global measures of glucose homeostasis by MMPI required 6 months in rats [9] and may be even longer in a more genetically mixed population of people. DM2 participants in this trial were on antihypertensive, lipid lowering and hypoglycemic medications which may have affected changes in insulin sensitivity. Larger numbers of participants with both new onset DM or impaired glucose tolerance or impaired fasting glucose with minimal medication use and a longer trial will be important in determining if there are any benefits to MMPI use in the treatment of inflammation and reduced insulin sensitivity in humans. If doxycycline were to be used long-term, studies on development of resistance or changes in gastrointestinal flora would be helpful to understand the contribution of these changes to markers of inflammation and insulin sensitivity. Additional limitations of this study include the lack of assessment of tissue MMPs, and small study size.

## Conclusions

Treatment of obese diabetics with doxycycline for 12 weeks significantly decreased serum markers of inflammation while increasing muscle tissue insulin sensitivity; these data suggest that longer studies in people should be explored. Studies with larger numbers of patients, longer treatment periods of escalating doses of doxycycline and a detailed evaluation of the underlying mechanisms leading to improvements in inflammation and insulin sensitivity may provide greater insight into the potential for using doxycycline and other MMP inhibitors in human disease.

## Abbreviations

MMP: Matrix metalloproteinase; PL: Placebo; MMPI: MMP inhibitor; DM2: Type 2 diabetes; PDK1: 3’phosphoinositide kinase-1; PKB/Akt: Protein kinase B; GSK3 ß: Glycogen synthase kinase 3ß; PI3K: Phosphatidyl inositol-3-kinase; GLUT4: Glucose transporter; CRP: C-reactive protein; MPO: Myeloperoxidase; OGTT: Oral glucose tolerance test; DXA: Dual-energy x-ray absorptiometry; A1c: Hemoglobin A1c; HOMA: Homeostasis model assessment.

## Competing interests

GWSS holds interest in InhibeX, a company that has licensed MMP treatment from UCSD.

## Authors’ contributions

KH, GWSS, FV conceptualized this study and developed the initial study structure. KH, KF, CT, and MB conducted subject visits, collected samples, and input data for analyses, statistics and writing the paper. RL, MT, and MH conducted MMP 2/9 activity data, conducted statistics and wrote methods for these data. MTG conducted muscle and MPO assays and wrote methods and results for these data. KF, KH, CT were involved in writing the paper including statistics and figure development. All authors read and approved the final manuscript.
